# Buckwheat Hull Extracts Inhibit *Aspergillus flavus* Growth and AFB_1_ Biosynthesis

**DOI:** 10.3389/fmicb.2019.01997

**Published:** 2019-08-29

**Authors:** Chiara Nobili, Agnese De Acutis, Massimo Reverberi, Cristiano Bello, Gian Paolo Leone, Domenico Palumbo, Fausta Natella, Silvia Procacci, Slaven Zjalic, Andrea Brunori

**Affiliations:** ^1^Department for Sustainability, ENEA, Rome, Italy; ^2^Department for Environmental and Evolutionary Biology, Sapienza University of Rome, Rome, Italy; ^3^AST Scienze della Nutrizione, Council for Agricultural Research and Economics (CREA), Rome, Italy; ^4^Department of Ecology, Aquaculture and Agriculture, University of Zadar, Zadar, Croatia

**Keywords:** *Aspergillus flavus*, biomolecules, *Fagopyrum esculentum*, industrial waste recovery, organic pest-control

## Abstract

Fungal contamination poses at risk the whole food production chain - *from farm to fork* - with potential negative impact on human health. So far, the insurgence of pathogens has been restrained by the use of chemical compounds, whose residues have gradually accumulated determining toxic effects in the environment. Modern innovative techniques imply the use of natural and eco-sustainable bioactive plant molecules as pathogens and pests-control agents. These may be profitably recovered in large amounts at the end of industrial milling processes. This is the case of the non-digestible hull of common buckwheat (*Fagopyrum esculentum* Moench), a natural source of polyphenols, tocopherols, phytosterols and fatty acids. We extract these compounds from the hull of buckwheat; apply them to *Aspergillus flavus* - aflatoxin producer - under *in vitro* conditions, checking their ability to inhibit fungal growth and aflatoxin biosynthesis. Moreover, a solvent free method implying the adoption of supercritical CO_2_ as solvent was set up to extract lipophilic molecules from the buckwheat’ hulls. Positive results in controlling fungal growth and aflatoxin biosynthesis let infer that the extracts could be further tested also under *in vivo* conditions.

## Introduction

At a time when the focus on the environmental issues is very high and the concepts of sustainability and circular economy are the key points of scientific community, researchers aim to find alternatives to synthetic pesticides whose use heavily affected the environment. The increasing yield losses associated to pests and diseases (up to 30% worldwide), and the expanding demand from the agro-food industry for a higher quality and availability of the products, led to a massive use of fertilizers, fungicides and synthetic pesticides (agropharmaceuticals) in the pre- and post-harvest (Janisiewicz and Korsten, 2002). Fungicides, and the effects determined by their use, normally receive minor attention compared to other types of agrochemicals such as insecticides and herbicides. It is a common belief that fungicides have a lower toxicity compared to other agrochemicals such as pesticides; however, recent studies demonstrated the opposite: i.e., fungicides revealed more toxic than herbicides and pesticides in a comparative assay (Mesnage et al., 2014). Indeed, chemicals used to control fungal contamination along the agro-food chain, leave residues that tend accumulating causing a negative impact in the environment (Liu et al., 2008; Wightwick et al., 2010; Abdallah et al., 2018). The increasing pollution can be reduced through the application of a more restrictive legislation, appealing to clearer rules to regulate the approval process for plant protection products (Directive 2009/128/EC) and to take them to the market (Regulation (EC) No 1107/2009), showing a great attention to environmental sustainability and consumer health.

In this scenario, molecules extracted from plants tissues could represent an alternative more sustainable than synthetic agrochemical. Such compounds have been successfully tested for applications in various sectors such as food industry, cosmetics and agro-chemistry (Balandrin et al., 1985; Miyakado, 1986; Benner, 1993; Hedin and Hollingworth, 1997) and, more recently, they have been utilized within systems of integrated pest management (El-Habib, 2012; Omidpanah et al., 2015). These natural extracts can possibly be recovered from waste materials through technologies with low environmental impact; thus favoring sustainability by promoting a “circular economy.” In the last decades emerged a growing interest for plant-derived bioactive substances possessing antioxidant, antibacterial and antifungal properties (Hussain et al., 2008; Venkateswarlu, 2013). In particular, the antimicrobial activity of polyphenols, such as flavonols, has been extensively investigated in various microorganisms, whilst, generally, antioxidant activities are associated to tocopherols, lipophilic compounds belonging to the vitamin E group (Daglia, 2012). Additionally, the antifungal effect of some lipid substances should not to be underestimated. Fatty acids and sterols can interact directly with the fungal cell membrane causing a generalized disorganization leading to changes in the growth pattern (Pohl et al., 2011).

The filamentous fungus *Aspergillus flavus* is widely spread; it affects a large number of plant species (e.g., maize, peanuts), with potential devastating consequences on yield and economic profits. Furthermore, *A. flavus* can produce aflatoxins, secondary metabolites that, in some instances, may result highly toxic (carcinogenic) for humans. Since now, the best strategy for controlling *A. flavus* resides on the field control measures provided by the use of antagonistic non-aflatoxigenic strains of the same species (Bhatnagar-Mathur et al., 2015; Mauro et al., 2018). It is possible to limit aflatoxin contamination at post-harvest level using some synthetic compounds (e.g., BHA) that nonetheless present their own toxicity (Nesci et al., 2003). In relation to this, it appears worth considering the possibility to control *A. flavus* and consequent aflatoxin production using plant-derived biomolecules. Similar compounds are present, in considerable amounts, in common buckwheat (*Fagopyrum esculentum* Moench) (Bonafaccia et al., 2003; Dziadek et al., 2016). The nutritional traits and the nutraceutical properties of buckwheat achenes have been studied and are well known (Soral-Smietana et al., 1984). Buckwheat grains are rich in phenolic compounds and tocopherols, fatty acids and phytosterols (Dorrell, 1971; Sedej et al., 2012). Among the various grain parts, the pericarp contains the highest amounts of these molecules (Dietrych-Szostak, 2004). Therefore, buckwheat hull, normally regarded in Europe as an industrial waste, may instead represent a low cost source of “beneficial” molecules, thus promoting the recycle of otherwise unusable exhausted raw materials.

To optimize for the sake of sustainability, the extraction of lipophilic biomolecules contained in buckwheat hull, an environmental-friendly extracting process, represented by the supercritical fluid extraction process using carbon dioxide (SFE-CO_2_) was adopted. Due to its non-polar nature, SFE-CO_2_ can easily solubilize lipophilic substances avoiding solvent contamination and chemical modification. Additionally, carbon dioxide has the advantage of being non-toxic, non-explosive, chemically inert, non-flammable, and inexpensive and, due to its volatility, at the end of the process, the extract can be considered as solvent-free (Raventós et al., 2002; Nautiyal, 2016).

In this study, we test - *in vitro* - lipophilic compounds - tocopherols, phytosterols and fatty acids - extracted using SFE-CO_2_; and polyphenols, i.e., isoorientin, vitexin, rutin, isovitexin, hyperoside and quercetin obtained from buckwheat hull, to evaluate their efficacy to inhibit the growth of *A. flavus* and the biosynthesis of aflatoxin B_1_ (AFB_1_).

## Materials and Methods

All chemicals were purchased from Sigma-Aldrich, United States.

### Buckwheat Hull

A mix of hulls resulting from the milling of three *F. esculentum* varieties (Bamby, Špačinská e Lileja), was provided by “GARFAGNANA COOP,” a small company located in Central Italy. For analytical purpose, buckwheat hull was grinded through a “CYCLOTEC 1093 Sample mill” (Tecator) and then sieved with <1000 μm mesh.

### Polyphenols Analysis

Sieved hull was freeze-dried, powdered in liquid nitrogen, extracted with a 80:20 ethanol:water solution (Morishita et al., 2007) and filtrated with a 0.20 μm PTFE filter.

Total polyphenols were quantified as described by Emmons et al. (1999), with some modifications. A total of 250 μL of extract were added with 4 mL of water, 500 μL of Na_2_CO_3_ and 250 μL of Folin-Ciocâlteau (1:1 v/v in water), and shaken. After 25 min of incubation in the dark at 25°C, samples were centrifuged at 5000 rpm for 10 min. The absorbance was detected at 725 nm by a “Beckman DU530 UV/VIS” spectrophotometer, using a 80:20 ethanol:water mixture as blank. Polyphenols content was determined in comparison to a calibration curve of Gallic acid (3,4,5-Tri-hydroxybenzoic acid) in the range between 10 and 500 ppm. All samples, in triplicates, were measured three times and results expressed as ppm (mg/L) of Gallic acid and data are presented as the means (±SD) of 3 separate experiments (*n* = 9).

The antioxidant activity was evaluated, in triplicates, as described in Chitarrini et al. (2014) and data are presented as the means (±SD) of 3 separate experiments (*n* = 9).

The chromatographic separation of isorientin, vitexin, rutin, isovitexin, hyporoside, and quercetin was performed by HPLC (Agilent 1260, United States) equipped with a 1260 Quat pump (Varian, United States), a 1260 DAD detector, an Infinity 1260 auto sampler (Agilent, United States), using a Supelco Ascentis C18 RP-Amide (25 cm × 4.6 mm, 5 μm) analytical column. The eluent mixture was composed of acetonitrile (solution A) and water (solution B) both acidified with 0.1% HCOOH. The flow rate was set to 1 mL/min, the column was thermostated at 30°C and the detection wavelength was set at 362 nm. The separations were performed using different solution A concentrations according to the following program: 20 min of linear gradient elution from 20 to 85%, 5 min of isocratic elution at 85%, 5 min of linear gradient elution from 85 to 20%, followed by 2 min of isocratic elution at 20%. The identification of individual compounds was performed based on their retention times and UV spectra. Libraries comprising retention times and UV-visible spectra were made by subjecting solutions of each standard. Using the Open LAB (Agilent, United States) software, a similarity index (SI) was calculated to evaluate how closer spectra of standard and corresponding phenolic compounds separated in the extracts resemble each other. According to the above software, SI closer to unity is indicative of higher similarity. In addition, the use of a purity index (PI), based on the comparison of all the spectra within a chromatographic peak to the spectrum at the peak apex, allowed one to exclude the presence of co-eluting substances in the peaks of the phenolic compounds separated from the extracts. Quantification of individual compounds was performed by calibration curves in the range of 50–1500 ng, using Kaempferol as internal standard (IS). Limit of detection (LOD) and limit of quantification (LOQ) were 5 and 11 ng, respectively. Recovery, performed adding spike compounds in amounts equal to 50, 100, and 150% of the measured analytes to lyophilized buckwheat hull, ranged between 81 and 97%, indicating a good accuracy of the method.

### Lipophilic Compounds Extract Analysis

A rough characterization of the lipid fraction contained in buckwheat hull was carried out according to Christie and Han (2010) in order to confirm the presence of compounds under investigation. This preliminary assay was performed comparing the retention factor (Rf) of the spots of the lipid fraction with the Rf of the spots of standard compounds developed in the same conditions.

The ascertained presence of the species of interest in significant amounts allowed proceeding with the extraction of lipophilic compounds with a Dionex SFE-703 counter extractor, using carbon dioxide as sole extracting solvent, at 300 bar, 40°C for 180 min. Furthermore, the extraction can be performed avoiding thermal degradation and reducing energy consumes, thanks to the associated low critical values (Tc = 31.08°C; Pc = 73.8 bar) compared to those showed by similar gases. The most appropriate process conditions were suggested by the published literature (Wang et al., 2008; Tomita et al., 2014) and preferring a particle size of less than 1 millimeter to improve the surface/volume ratio to favor the diffusion process for extraction of the solute from inside the solid phase.

To verify the efficiency of SFE-CO_2_ method, the pool of tocopherols, phytosterols and fatty acids was in parallel extracted with organic solvents, hereinafter reported as “conventional extraction.”

Tocopherols and phytosterols conventional extraction was performed according to Slavin and Yu (2012), with minor modifications, and data are presented as the means (±SD) of three separate experiments (*n* = 9). Aliquots (1 g) were weighed and added with 250 μg of α-tocopherol acetate acting as internal standard. This mixture was added with 3 mL of an ethanolic solution containing 0.1% (w/v) of tert-Butyl hydroperoxide (TBH) and shaken for 10 s. The sample obtained was, in sequence, placed in a thermostatic bath at 85°C for 5 min, added with 190 μL of a 10 M potassium hydroxide solution (KOH), shaken for 10 s, incubated at 85°C for 10 min and finally cooled in ice for 10 min after the addition of 3 mL of NaCl 1M. Hexane extraction was carried out two consecutive times: samples were added with 3 mL of hexane, shaken for 10 s and centrifuged at 1000 rpm for 5 min at 4°C. The supernatant of the two extractions was combined in a new test tube, to be added with 5 mL of a 5% (w/v) Na_2_CO_3_ solution and subsequently centrifuged at 1000 rpm for 5 min at 4°C. The novel supernatant was washed with 5 mL of ultra-pure water and transferred to a clean test tube. The sample thus obtained was evaporated under nitrogen stream at room temperature. Separation, identification and quantification of α-, β-, γ-, δ-tocopherols, campesterol and β-sitosterol was performed by HPLC (Perkin Elmer Series 200) coupled to a mass spectrometer (AB Sciex QTrap 3200) equipped with Atmospheric Pressure Chemical Ionization source (APCI) in Multiple Reaction Monitoring (MRM). After appropriate dilution, the samples were separated by XBridge Phenyl column 150 × 2.1 mm, 3.5 μm (Waters), thermostated at 25°C with a flow of 0.25 mL/min. The mobile phases were as follows: Phase A containing H_2_O acidified with 0.1% formic acid, Phase B containing acetonitrile acidified with 0.1% formic acid. Separation was performed by isocratic elution with phase B at 90%. The injection volume was 10 μL. The MS/MS acquisition was performed using an APCI source optimized with the following parameters: Curtain Gas (CUR) 40, Temperature Source (TEM) 400°C, Spray Gas (GS1) 30, Heater Gas (GS2) 30, Spray Current (NC) 5 mA, Entrance Potential (EP) 7 kV, dwell time 100 msec. Declustering Potential (DP) and Collision Energy (CE) were optimized for single transitions in MRM mode: α-tocopherol m/z 431/165, 431/137, DP 30, CE 20; β and γ-tocopherol, m/z 417/191, 417/151, DP 30, CE 25; campesterol, m/z 383/161, DP 30, CE 20; β-sitosterol, m/z 397/161, DP 30, CE 30, m/z 397/135, DP 30, CE 35; ergosterol, m/z 379/69, DP 30, CE 25; stigmasterol, m/z 395/297, DP 30, CE 15, m/z 395/83, DP 30, CE 35; δ-tocopherol, m/z 403/177, 403/137, DP 25, CE 25. α-tocopherol acetate was used as internal standard (IS) m/z 473/431, 473/207, DP 30, CE 20. Acquisition and processing of data was carried out using Analyst 1.5.1 software. The analytic identification was performed by comparing retention times and MRM transitions of a standard mix. The quantification of the analytes was performed with a calibration curve in the linear range of 0.1–10 ng. LOD and LOQ were 0.002 and 0.006 ng, respectively. Recovery results ranged between 85 and 97%, indicating a good accuracy of the method.

The extraction of fatty acids was carried out according to Phippen et al. (2006), with slight modifications, and data are presented as the means (±SD) of three separate experiments (*n* = 9). Grounded hulls (300 mg) were weighed in a 25 mL Pyrex flask with cap, added with 2 mL of 0.5 M KOH in methanol, stirred for 1 min and placed in a water bath at 60°C for 60 min. At this stage, the flask was further incubated at 60°C for 15 min following the addition of 2 mL of a 1 M solution of H_2_SO_4_ in methanol. Then the sample was added with 2 mL of ultrapure water, left cooling for 10 min and finally added with 2 mL of hexane containing non-methylated C17: 0 (margaric acid) as internal standard. Supernatant (1 mL) was collected and dried under nitrogen stream at room temperature. Fatty acids were analyzed by gas chromatography (GC) 7890 B (Agilent, United States) equipped with: an injector set at 250°C, 20.443 psi, flow rate 28.2 mL/min, purge flow 3 mL/min, split 1:2 with flow 24 mL/min; OmegawaxTM 250 silicon capillary column (30 m × 0.25 mm df 0.25 μm, Supelco Analytical) with flow 1.2 mL/min, 20.443 psi, speed 33.995 cm/s, hold time 1.4725 min, post run 0.78872 mL/min; oven at 170°C, rate 1°C/min and Tf 230°C in 16 min. The injected volume and run time were set at 1 μL and 16 min, respectively. Chromatographic peaks were identified comparing retention times of a standard mixture of 37 fatty acids including: palmitic acid (C16:0), stearic acid (C18:0), oleic acid (C18:1), linoleic acid (C18:2) and linolenic acid (C18:3). Once identified, the amounts of these five compounds were quantified using a second more specific standard mixture (F.A.M.E. RM-2) containing the above-mentioned standards and margaric acid as internal standard. The identification of individual compounds was performed based on their retention times and the application of the standard addition method (Wrona et al., 2013). Quantification of individual compounds was performed via the external standard method. Linearity was evaluated based on the calibration curves that were constructed by plotting the concentration of standards in μg/mL versus peak area. Linear least-squares regression analysis was employed to calculate slope, intercept, and correlation coefficient of the calibration curve. This last parameter resulted higher than 0.9998 for all analytes, indicating good linearity, verified in the range 7–70 ng for palmitic acid (C16:0), 5–50 ng for stearic acid (C18:0), 18–180 ng for oleic acid (C18:1), 35–358 ng for linoleic acid (C18:2) and 34–340 ng for linolenic acid (C18:3). Limits of detection (LOD) and limits of quantification (LOQ), were 0.8, 0.6, 1.9, 3.6, 3.5 and 2.1, 1.2, 3.8, 17.5, 17.1 ng respectively. Recoveries varied between 83 and 95%, thus proving an adequate degree of accuracy.

### Evaluation *in vitro* of the Antifungal Activity of Extracts on *A. flavus* Growth

AFB_1_-producing *A. flavus* strain - NRRL 3357 was maintained on Czapek Dox Agar (CDA), amended with ZnSO_4_ (5 mg/L) and NaMoO_4_ (1 mg/L), at 30°C. Extract fraction containing polyphenols (PE), and lipophilic compounds obtained with supercritical CO_2_ (LE) were tested to verify the ability to modulate either mycelial growth or AFB_1_ production at different time intervals: 4, 5, 6, and 7 days after inoculation (DAI). PE and LE concentrations showing higher inhibitory ability were determined after considering several combinations. In order to evaluate antifungal activity of buckwheat hull extracts on *A. flavus* growth, 5 Petri dishes (100 mm × 15 mm size) for each experiment, containing potato Dextrose Agar (PDA), amended with either PE (100 ng/mL), LE (10 μg/mL) or a mixture of both, were inoculated with a pure culture of the fungus. A positive control (ctr+), inoculated but not containing extracts and a non-inoculated negative control (ctr−) were also included. The incubation temperature was set up at 30°C for 7 days, until the appearance of mature spores according to Liu et al. (2016) and Alshannaq et al. (2018). At the end of the incubation period (7 DAI), mean radial mycelial growth was determined by measuring the size of the colony at two perpendicular directions and presented as the means (±SD), while for multiple comparison analysis (Tukey’s test) on fungal growth inhibition (%), the mean growth values were recorded and compared to the control treatment to determine the mycelial growth inhibition (MGI) percentage through the formula,

MGI(%)=((d-cd)t/d)c×100

where d_*c*_ and d_*t*_ represent mean mycelial growth size in control and treated Petri dishes, respectively.

### Evaluation *in vitro* of the Antifungal Activity of Extracts on AFB_1_ Production

Phenolic and lipophilic (LE) extracts’ influence on aflatoxin B_1_ production was evaluated on *A. flavus* grown on potato dextrose broth (PDB) over a period of 7 days and data are presented as the means (±SD) of five separate experiments. Beside different concentrations of PE (50, 100, 500 ng/mL) and LE (1, 10, 100 μg/mL) also the following combinations (PE 50 ng/mL and LE 1 μg/mL, PE 50 ng/mL and LE 10 μg/mL, PE 100 ng/mL and LE 10 μg/mL) were evaluated. Positive control (ctr+) adopted was extract free, whereas not inoculated growth media served as negative control (ctr−). AFB_1_ determination was performed as previously reported (Fanelli et al., 2004), by extracting *A. flavus* culture in chloroform:methanol (2:1 v/v) three times. The extracts were collected after filtration on anhydrous Na_2_SO_4_ and concentrated under a N_2_ stream. AFB_1_ quantification was performed by HPLC (Agilent 1260, United States) equipped with a 1260 Quat pump (Varian, United States), a 1260 DAD detector, an Infinity 1260 auto sampler (Agilent, United States); using an analytical column GEMINI^®^ C18 (LC Column, 150 mm × 4,6 mm, 5 μm, 110 Å, Phenomenex). Mobile phase was a mixture of water/acetonitrile (70:30 v/v). The flow rate was set to 1 mL/min, the column was thermostated at 40°C and the detection wavelength was 363 nm. Crystalline AFB_1_ was used to prepare the standard solution. AFB_1_ content was calculated on the basis of the calibration curve, 25–1000 ng of AFB_1_ standard. Quantification of AFB_1_ was performed by the external standard method. Linearity was evaluated on the basis of the calibration graphs that were constructed by plotting the concentration of standards 25–1000 ng of AFB_1_ in μg/mL versus peak area. Linear least-squares regression analysis was employed to calculate slope, intercept, and correlation coefficient of the calibration graph. The correlation coefficient of the calibration graph was higher than 0.9978, indicating good linearity. The linearity was verified in the range 15–500 ng. Limit of detection (LOD) and (LOQ) were 1.6 and 8.2 ng respectively. The accuracy of the method was evaluated by a recovery study, which was carried out according to the procedure afore mentioned for polyphenols. The recoveries were between 88.2 and 103.8%, indicating that the method has an adequate degree of accuracy. Data are presented as the means (±SD) of five separate experiments, while for multiple comparison analysis (Tukey’s test) on inhibition of AFB_1_ production; the mean values were normalized to the control treatment (ctr+).

### Statistics

To verify if MGI and AFB_1_ synthesis inhibition raw data means were significantly different from each other, a multiple comparison test was performed on the information returned by ANOVA test using Tukey’s test honestly significant difference procedure. As it is known, two group means are significantly different if their comparison intervals are disjoint (we assumed a significance level of 0.05). In fact, values which present not overlapping errors bars are significantly different (*ρ* < 0.05). Correlations of variables, MGI and AFB_1_ inhibition, were suggested by the scatter plot. All the statistical analyses were performed using MATLAB R2015b software.

## Results and Discussion

### Buckwheat Hull Phenolic Content Analysis

The content in term of total polyphenols found in buckwheat hull is 4.89 mg/g Dry Weight-DW, confirming an antioxidant activity equal to 7.41 μmol Trolox EQ/g, in line with values previously associated with antifungal effect (Alvarez-Jubete et al., 2010; Żmijewski et al., 2015). Polyphenols extracted (PE) from buckwheat hull can alter the antioxidant system of the fungal cells, although temporarily, and are the main responsible for the inhibition of AFB_1_ production as well as of fungal growth (Nesci et al., 2003).

Amongst the investigated polyphenols, vitexin and hyperoside are present in a significantly higher concentrations within the hulls (Table 1 and Supplementary Figure S1), with average values of 426 and 440 μg/g, respectively. Concentration of rutin, the polyphenol, characterized by the highest antioxidant activity among those investigated (Dietrych-Szostak and Oleszek, 1999), was 206 μg/g DW, value similar to those observed in buckwheat grain (Brunori et al., 2010).

**TABLE 1:** Contents (μg/g) of polyphenols in buckwheat hull extract.

	**μg/g**
Isoorientin	247.3 ± 5.2
Vitexin	425.7 ± 9.1
Rutin	206.0 ± 4.8
Isovitexin	269.1 ± 5.0
Hyperoside	440.1 ± 9.3
Quercetin	39.1 ± 0.7

*Values are reported as means ± SD.*

These evidences are in agreement with the current literature which reports that phenolic compounds are potent antioxidants showing often antifungal activity, e.g., against *Aspergillus* (Hua et al., 1999; Kim et al., 2005, 2006; Razzaghi-Abyaneh et al., 2008). Moreover, polyphenols may exert inhibitory activity on AFB_1_ production modulating oxidative stress levels in the fungal cell (Bhatnagar et al., 2008; Kim et al., 2008; Brown et al., 2010). Reactive Oxygen Species (ROS) may regulate all the vitally important processes in fungi: phase development change, intercellular communications, and protection from interspecies competition proving to be a prerequisite and stimulatory factor for AFB_1_ biosynthesis (Jayashree and Subramanyam, 2000; Zaccaria et al., 2015). At physiological concentrations, ROS play an important role in fungal developmental processes (Reverberi et al., 2008), but if their level exceeds the cell-scavenging capacity, cell membranes and cell metabolism may result damaged. Interestingly, the same effect can be obtained also with low ROS concentrations. Those seem likely to suppress the spore germination at a regulatory level (Gessler et al., 2007; Breitenbach et al., 2015).

### Lipophilic Extract Characterization

**FIGURE 1:**
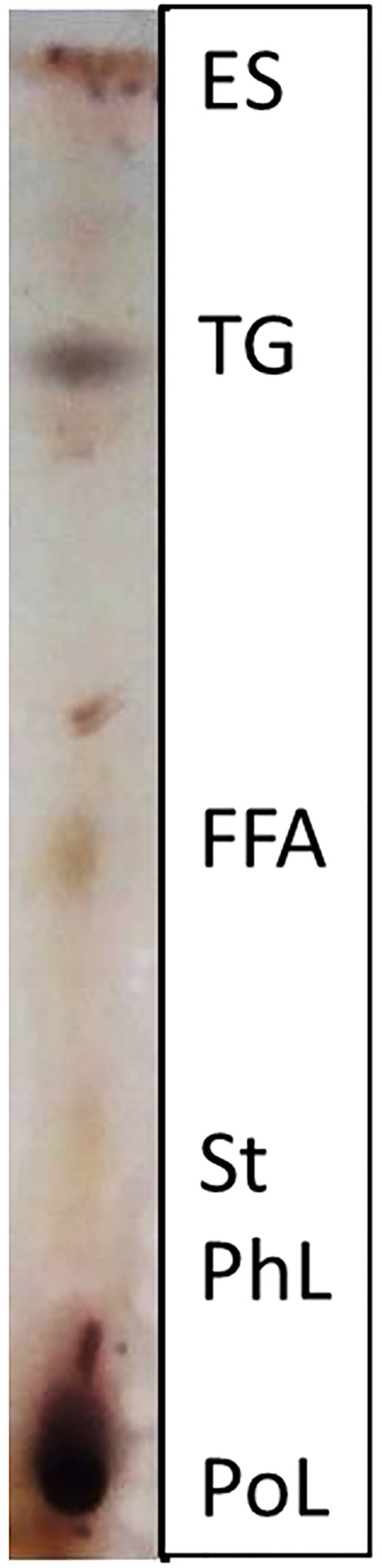
Thin layer chromatograply (TLC) analysis of lipid fraction. PoL, polar lipids; PhL, phospholipids; St, sterols; FFA, free fatty acids; TG, triglycerides; ES, esters.

In the present study, TLC analysis of the lipid fraction (LE) of buckwheat hull indicated high levels of polar lipids (PoL), phospholipids (PhL), sterols (St) and free fatty acids (FFA), and low amounts of triglycerides (TG) and sterol esters (ES) (Figure 1). Tocopherols, fatty acids and phytosterols extraction was performed with both conventional protocols and supercritical CO_2_ fluid extraction (SFE-CO_2_; Supplementary Table S1), which enables yield increase (up to three orders of magnitude). This extraction method proved to be more conservative favoring the preservation and augmenting the yield of lipophilic compounds (Table 2; Uquiche et al., 2015).

**TABLE 2:** Contents (μg/g) of tocopherols, sterols and fatty acids in hull extract obtained using conventional extractions or supercritical CO_2_ as eluent.

	**Conventional extractions**	**CO_2_ extraction**
α- tocopherol	144 ± 5	100 ± 4
δ-tocopherol	−	19.5 ± 0.6
γ-tocopherol	16.9 ± 0.5	602 ± 24
β-sitosterol	384 ± 12	9474 ± 452
campesterol	40.5 ± 0.4	1678 ± 51
stigmasterol	158 ± 5	5686 ± 194
C16:0	551.1 ± 9.1	378.3×10±34.7×103
C18:0	56.6 ± 0.9	56.6×10±30.8×103
C18:1	528.2 ± 9.2	196.1×10±35.1×103
C18:2	515.9 ± 9.2	193.4×10±35.3×103

*Values are reported as means ± SD.*

Compounds such as tocopherols, fatty acids and phytosterols gain relevance in fungal growth and mycotoxin synthesis inhibition processes (Gonelimali et al., 2018). Specifically, antifungal activities of non-polar compounds involve the alteration of the cell membrane; this, in fungi, may lead to a loss of membrane permeability and to the accumulation of toxic substances, which disrupt the cell metabolism and activate the cytolytic pathways (Pohl et al., 2011; Sedej et al., 2012).

In plant tissues, the main function of tocopherols - the most important and abundant lipid-soluble antioxidant - is to reduce the amount of Reactive Nitrogen Oxide Species (RNOS), capable in turn to induce oxidative stress (Falk and Munnè-Bosch, 2010). In plants, α-tocopherol deactivates photosynthesis-derived reactive oxygen species, and prevents the propagation of lipid peroxidation by scavenging lipid peroxyl radicals in thylakoid membranes (Munné-Bosch, 2005). Γ-tocopherol, an isomer of vitamin E, is regarded as the most potent free-radical remover, able to detoxify electrophiles, thanks to its ability to form a stable nitro adduct, 5-Nγ-tocopherol (Cooney et al., 1993; Jiang et al., 2001). Conversely, γ-tocopherol is very unstable and at risk of denaturation under “conventional extraction” conditions (Sabliov et al., 2009). This may be prevented when a more conservative method such as supercritical fluid extraction (SFE-CO_2_) is adopted. Furthermore, concerning *A. flavus*, tocopherols have a high affinity to aflatoxin, reducing its bioavailability through the formation of stable associations (Daud et al., 2014).

Phytosterols, as well, express their toxicity against fungi via interference with cell membrane integrity (Augustin et al., 2011; Singh et al., 2016), playing an important role in fundamental biological processes like signal transduction, cellular sorting, cytoskeleton reorganization and infection process (Simons and Ikonen, 2000; Simons and Ehehalt, 2002). Regarding β-sitosterol, it has been reported that its exogenous application may favor an increased resistance to fungal diseases, presumably in connection with the loss of membrane integrity of the plant pathogens (Siebers et al., 2016).

Free fatty acids lipid fraction can cause an elevation in fluidity of cell membrane increasing the mobility of phospholipid acyl chains in the membrane bilayer in proportion with their degree of unsaturation (18:1 > 18:2 > 18:3) (De Kruyff et al., 1973; Avis and Bèlanger, 2001). Among other, oleic and linoleic acids, which resulted the most abundant in the lipophilic extract (LE), are known to express a strong antifungal activity either on the spore germination, on the mycelial growth or both depending on the fungus lifestyle (Altieri et al., 2007; Liu et al., 2008). Palmitic acid, the other fatty acid present in high concentration, is characterized by an antifungal activity quite higher compared to unsaturated fatty acids (Liu et al., 2008).

### Evaluation *in vitro* of Buckwheat Hull Extracts Influence on *A. flavus* Growth and AFB_1_ Synthesis

**FIGURE 2:**
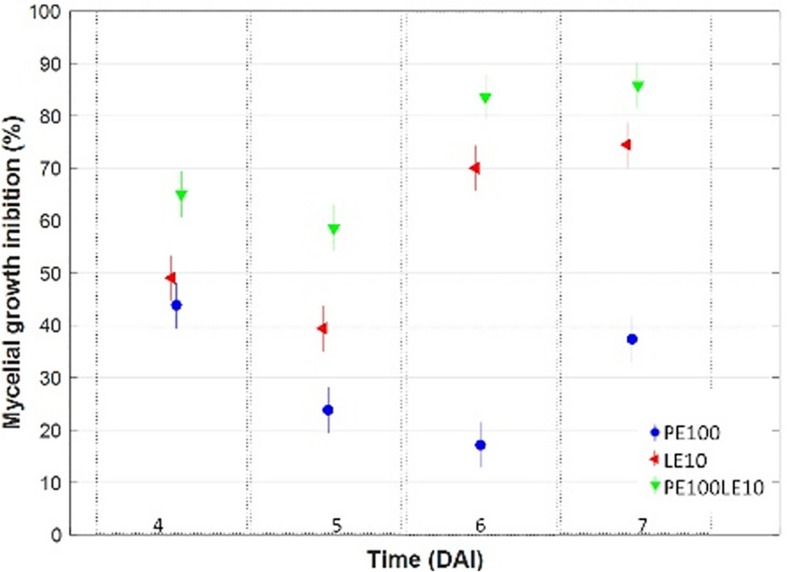
Multiple comparison test (with Tukey’s honestly significant difference procedure) on mycelial growth inhibition (%) trend occurred in growth media amended with PE (100 ng/mL), with LE (10 μg/mL) and a mix of both, at different days after inoculation (DAI).

To investigate the effectiveness of the buckwheat extracts against *A. flavus* mycelial growth under *in vitro* conditions, the lipid fraction (LE) obtained with SFE-CO_2_ was added to the culture medium, at 10 μg/mL and 100 ng/ml, respectively, either alone (LE10; PE100) or in mixed combination (LE10PE100) with polyphenol extract (PE), (Table 3 and Supplementary Figure S2). Raw data were normalized with the control obtaining a growth-inhibition trend (Figure 2). Despite the different timeline of the trends, the most evident inhibition was expressed by the combination of the two extracts (PE100LE10) in every time interval. By the end of the incubation period (7 DAI), *A. flavus* growth was reduced, respectively by 74 and 38% following LE10 and PE100 application, whereas the combination of the two fractions, determined a mycelial growth reduction of the 86%.

**TABLE 3:** Fungal growth (cm) in cultural medium amended with phenolic extract (PE) and lipophilic extract (LE), monitored: 4, 5, 6 and 7 days after inoculation (DAI).

	**4 DAI**	**5 DAI**	**6DAI**	**7 DAI**
ctr+	1.06 ± 0.05^A^	1.41 ± 0.07^A^	5.06 ± 0.24^A^	9.93 ± 0.49^A^
PE 100 ng/mL	0.63 ± 0.03^B^	1.08 ± 0.06^B^	4.15 ± 0.21^B^	6.21 ± 0.31^B^
LE 10 μg/mL	0.51 ± 0.03^B^	0.86 ± 0.04^BC^	1.54 ± 0.08^C^	2.58 ± 0.13^C^
PE 100 ng/mL+ LE 10 μg/mL	0.35 ± 0.02^B^	0.58 ± 0.03^C^	0.86 ± 0.05^D^	1.42 ± 0.07^D^

*Values are reported as means ± SD. Negative control (ctr−) and positive control (ctr+) corresponded, respectively, to non-inoculated growth medium amended with extracts, and infected growth medium without extracts. Small capital letter in the charts represent the significantly different groups (*p* < 0.05; Tukey test).*

**FIGURE 3:**
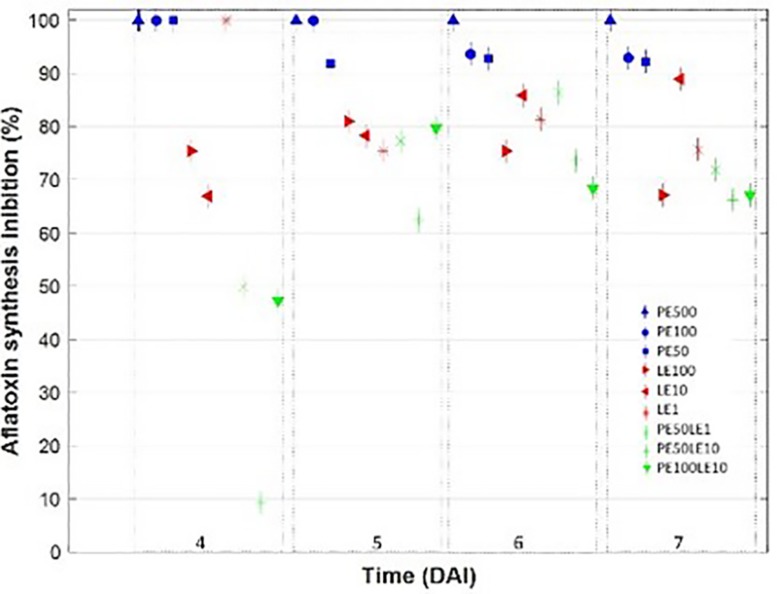
Multiple comparison test (with Tukey’s honestly significant difference procedure) on AFB_1_ synthesis inhibition (%) trend occurred in growth media amended with PE, LE and a mix of both in different concentrations, at different days after inoculation (DAI).

To outline the ability of PE and LE to inhibit AFB_1_ biosynthesis in *A. flavus*, a wide range (Table 4 and Supplementary Figure S3) of concentrations was tested as single as in combination. Notably, PE 500 ng/mL totally inhibited AFB_1_ synthesis during the period of observation, while PE 50 and 100 ng/mL inhibition lasted up to 4 and 5 DAI, respectively (Figure 3). This evidence suggests that, at least for the PE fraction, the inhibition of AFB_1_ production is dose-dependent: the higher the concentration the longer the inhibition.

**TABLE 4:** Amount of aflatoxin B_1_ (ng/mL) produced in growth medium amended with phenolic extract (PE) and lipophilic extract (LE) at different concentrations.

	**4 DAI**	**5 DAI**	**6 DAI**	**7 DAI**
ctr +	344.37 ± 17.28^A^	951.94 ± 46.8^A^	1479.47 ± 73.6^A^	2207.29 ± 109.5^A^
PE 500 ng/mL	0^F^	0^F^	0^G^	0^E^
PE 100 ng/mL	0^F^	0^F^	93.01 ± 4.65^F^	157.01 ± 7.26^D^
PE 50 ng/mL	0^F^	77.33 ± 3.48^E^	107.54 ± 5.34^F^	170.60 ± 8.45^D^
LE 100 μg/mL	84.50 ± 4.22^E^	180.67 ± 9.15^D^	364.23 ± 18.84^C^	725.45 ± 35.72^B^
LE 10 μg/mL	113.40 ± 5.62^D^	207.44±10.42DC	209.41 ± 11.01^E^	244.15 ± 12.94^D^
LE 1 μg/mL	0^F^	233.45 ± 11.75^C^	274.90 ± 13.81^D^	535.01 ± 26.88^C^
PE 50 ng/mL + LE 1 μg/mL	172.28 ± 8.59^C^	216.16 ± 10.79^CD^	200.68 ± 10.82^E^	619.10 ± 29.61^C^
PE 50 ng/mL + LE 10 μg/mL	311.88 ± 14.7^B^	356.79 ± 18.84^B^	389.97 ± 19.59^C^	742.48 ± 36.98^B^
PE 100 ng/mL + LE 10 μg/mL	180.89 ± 9.12^C^	192.07 ± 9.53^D^	465.77 ± 23.56^B^	726.72 ± 34.87^B^

*Values are reported as means ± SD. AFB_1_ production was monitored: 4, 5, 6, and 7 DAI. Negative control (ctr−) and positive control (ctr+) corresponded, respectively, to non-inoculated growth medium amended with extracts, and infected growth medium without extracts. Small capital letter in the charts represent the significantly different groups (*p* < 0.05; Tukey test).*

Lipophilic application showed over time a lower and apparently not dose dependent inhibitory effect on AFB_1_ synthesis. In general, no trend was evident except in the case of LE10, where the effect was continuosly increasingly with time. A peak value of 100% was observed for threatment of LE1 on 4 DAI followed by a rapid decrease, whereas the best performing threatment resulted LE10 with an average inhibition > 80% (Table 4). The behavior of the PE-LE mixtures appeared independent from the concentrations of the two fractions leading to a significant inhibition of AFB_1_ biosynthesis attesting around 70%. Actually, at 7 days after inoculation, PE, regardless the concentration tested, showed an inhibitory effect on AFB_1_ production significantly higher (>90%) compared to the other threatments. As regards LE, the highest inhibition, around 85%, was attained at 10 μg/mL, while any of the mixtures containing both lipofhilic fraction and poliphenols pooled around 70%. Nevertheless, none of the mixtures tested proved to inhibit AFB_1_ production as much as the sole PE.

**FIGURE 4:**
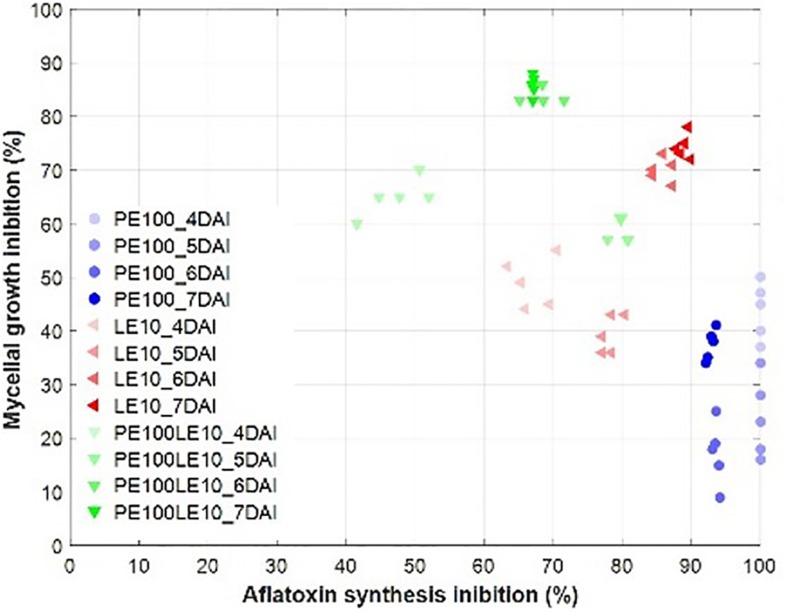
Scatter plot between mycelial growth inhibition (%) and AFB_1_ synthesis inhibition (%) for PE (100 ng/mL), LE (10 μg/mL) and their mix from 4 to 7 DAI.

We draw a scatter plot for evaluating the combined ability of buckwheat hull extracts to limit the mycelium growth and the aflatoxin biosynthesis (Figure 4). The best synergic effect on both variables occurred in the presence of LE10 followed by PE100LE10 at 7 and 6 DAI, while PE alone acted almost exclusively on the aflatoxin synthesis reduction (>90%), inhibiting only up to 45% the fungal growth.

Results suggest the existence of important physiological mechanisms that induces PE and LE to counteract the mycotoxin production and to modulate the mycelial growth.

## Conclusion

This study demonstrates that buckwheat hull extracts - rich in polyphenols and lipophilic molecules - can be used for limiting *A. flavus* growth and AFB_1_ synthesis (Liu et al., 2008; Reverberi et al., 2012). It was observed, in fact, that the mixture of both extracts had the highest influence on fungal growth, while polyphenols exert their main effect on the production of AFB_1_.

When “mild technologies” (extraction with supercritical CO_2_) were applied, such compounds were extracted at a concentration significantly higher compared to conventional methods, with the further advantage that molecules of interest are solvent-free, suitable for open field applications with highly reduced potential risk for plants, operators and the environment.

The capacity demonstrated by active natural molecules extracted from a plant waste partly using an eco-friendly extraction technique could design a new strategy to counteract fungal contamination at field or storage level.

## Data Availability

All datasets generated for this study are included in the manuscript and/or the Supplementary Files.

## Author Contributions

CN and MR designed the study. CN, ADA, CB, GL, and SP performed the experiments. CN and DP analyzed the data. CN, AB, and MR wrote the manuscript. All authors reviewed the manuscript.

## Conflict of Interest Statement

The authors declare that the research was conducted in the absence of any commercial or financial relationships that could be construed as a potential conflict of interest.

## Supplementary Material

TABLE S1 Overview of analytical methods used to extract lipid fraction.

FIGURE S1 HPLC-DAD (Agilent 1260, United States) chromatograms of polyphenols in PE extracts, identified by mean of their DAD profile and compared with standards as well as published spectra.

FIGURE S2 Fungal growth (cm) in cultural medium amended with phenolic extract (PE) and lipophilic extract (LE), monitored at 4, 5, 6, and 7 days after inoculation (DAI). Positive control (ctr+) corresponded, respectively, to non-inoculated growth medium amended with extracts, and infected growth medium without extracts. Values represented the mean of *n* = 5 determinations ± SE.

FIGURE S3 AFB1 biosynthesis in cultural medium amended with phenolic extract (PE) and lipophilic extract (LE), alone or in combinations of a range of concentrations (indications provided in the graph legend) monitored at 4, 5, 6, and 7 days after inoculation (DAI). Positive control (ctr+) corresponded, respectively, to non-inoculated growth medium amended with extracts, and infected growth medium without extracts. Values represented the mean of *n* = 5 determinations ± SE.
