# Supplementary material for: Buckwheat Hull Extracts Inhibit Aspergillus flavus Growth and AFB1 Biosynthesis
Source: Front Microbiol. 2019 Aug 29;10:1997. doi: 10.3389/fmicb.2019.01997 (PMC6727613; doi:10.3389/fmicb.2019.01997)
Supplement: TABLE S1 — Overview of analytical methods used to extract lipid fraction. [file Table_1.DOCX]

**Supplementary Table S1.** Overview of analytical methods used to extract lipid fraction.

| **Mild technology** |  | **Conventional extractions** |
| --- | --- | --- |
| SFE-CO_2_ | **Lipid fraction** | Christie and Han, 2010 |
|  | **Tocopherols:**  α-, β-, γ-, δ. | Slavin and Yu, 2012 |
|  | **Phytosterols**:  Campesterol,  β-sitosterol. |  |
|  | **Free fatty acids**:  palmitic acid (C16:0),  stearic acid (C18:0),  oleic acid (C18:1),  linoleic acid (C18:2),  linolenic acid (C18:3). | Phippen *et al*., 2006 |
