# Supplementary material for: Buckwheat Hull Extracts Inhibit Aspergillus flavus Growth and AFB1 Biosynthesis
Source: Front Microbiol. 2019 Aug 29;10:1997. doi: 10.3389/fmicb.2019.01997 (PMC6727613; doi:10.3389/fmicb.2019.01997)
Supplement: FIGURE S1 — HPLC-DAD (Agilent 1260, United States) chromatograms of polyphenols in PE extracts, identified by mean of their DAD profile and compared with standards as well as published spectra. [file Data_Sheet_1.PDF]

Current Chromatogram(s)

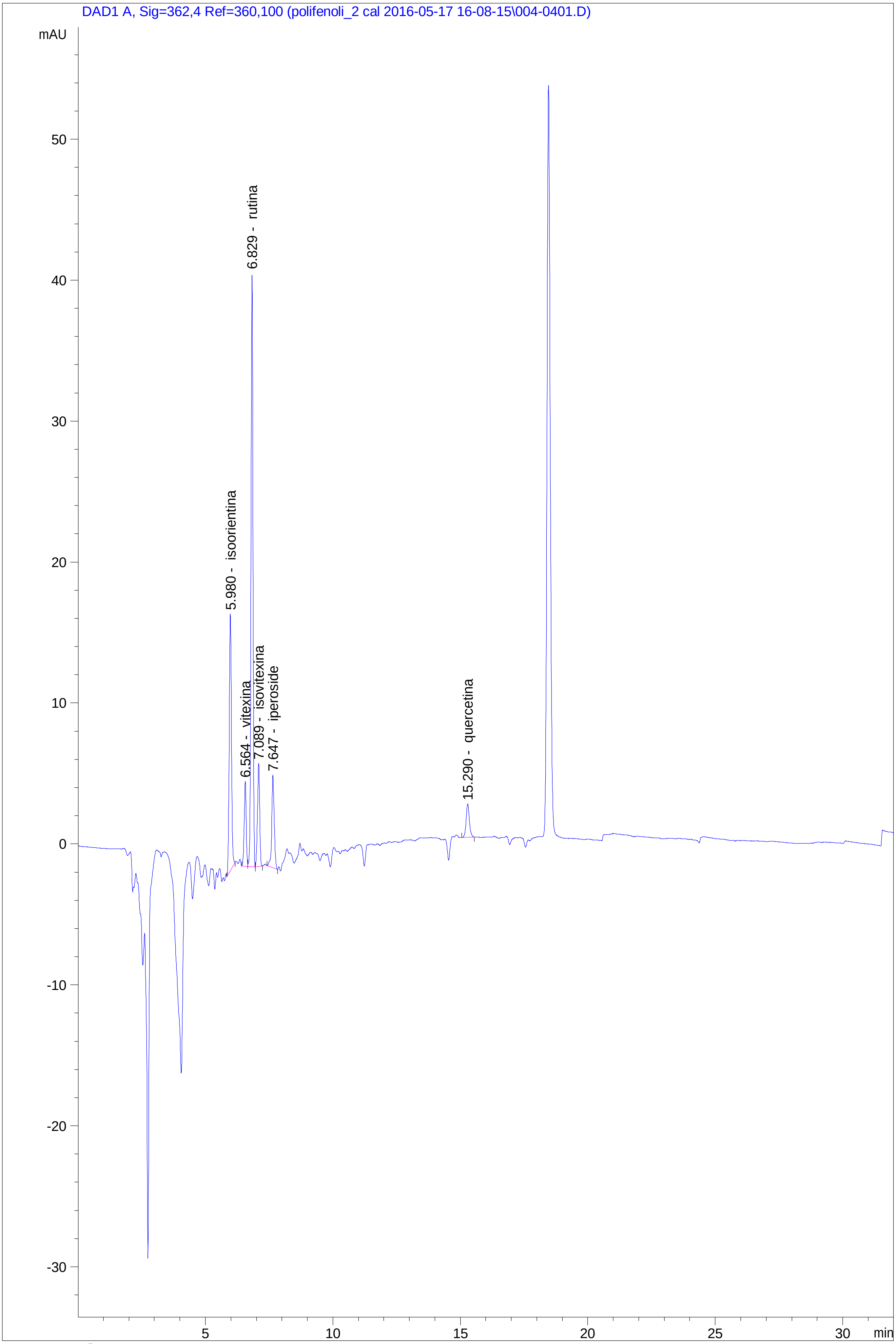

Data File C:\Chem32\1\Data\polifenoli_2 cal 2016-05-17 16-08-15\004-0401.D
Sample Name: 4

===

Acq. Operator : SYSTEM Seq. Line : 4
Acq. Instrument : HPLC 1260 Location : 4
Injection Date : 17-May-16 5:48:29 PM Inj : 1
Inj Volume : 20.000 µl
Acq. Method : C:\Chem32\1\Data\polifenoli_2 cal 2016-05-17 16-08-15\POLIFENOLI_2cal.M
Last changed : 17-May-16 4:08:15 PM by SYSTEM
Analysis Method : C:\Chem32\1\Data\polifenoli_2 cal 2016-05-17 16-08-15\POLIFENOLI_2cal.M (Sequence Method)
Last changed : 18-May-16 11:09:57 AM by SYSTEM
Method Info : Ascentis RP Amide 25 cm x 4,6 mm

Additional Info : Peak(s) manually integrated

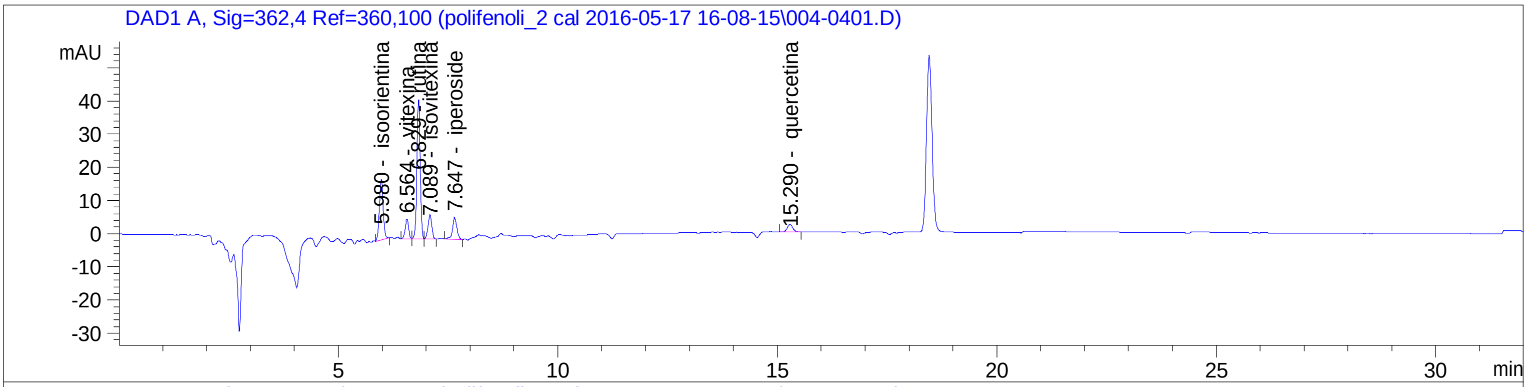

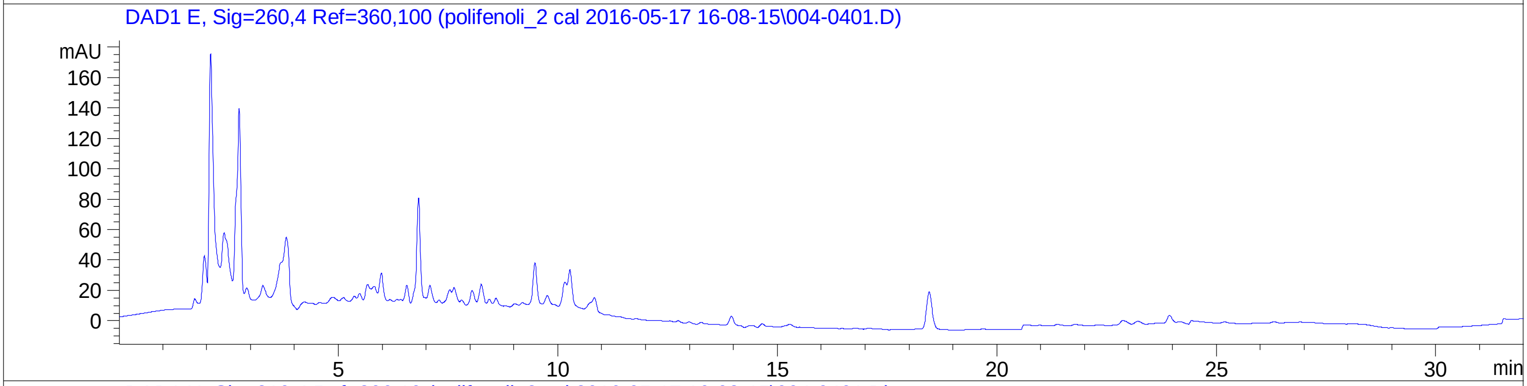

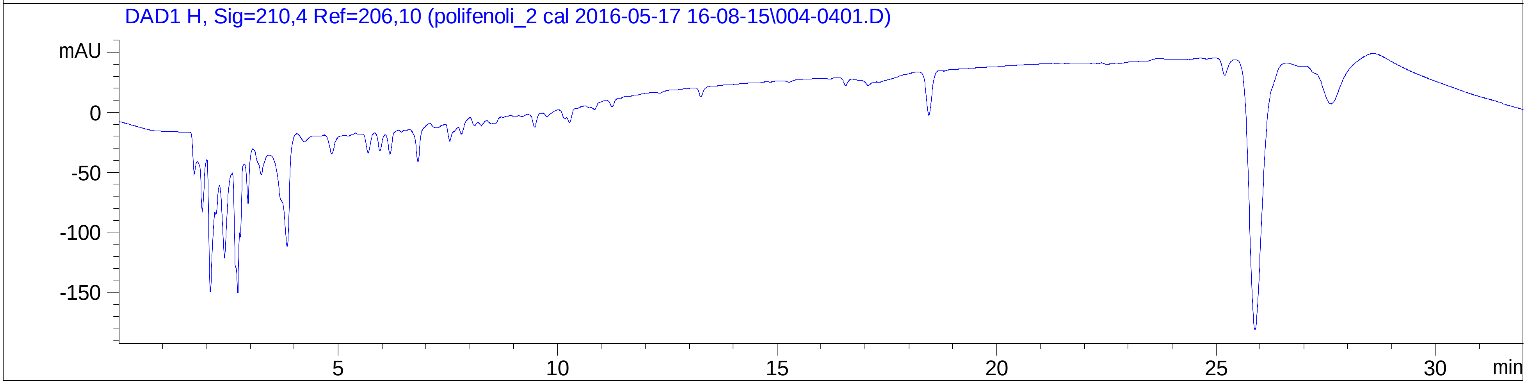

===

Internal Standard Report

===

Sorted By : Signal
Calib. Data Modified : Wednesday, May 18, 201611:04:34 AM
Multiplier : 1.0000
Dilution : 1.0000
Do not use Multiplier & Dilution Factor with ISTDs
Sample ISTD Information:
ISTD ISTD Amount Name

#	[ng/ul]	
1	1000.00000	kamferolo

Signal 1: DAD1 A, Sig=362,4 Ref=360,100

RetTime [min]	Type	ISTD used	Area [mAU*s]	Amt/Area ratio	Amount [ng/ul]	Grp	Name
5.980	BB	1	108.57949	0.00000	299.42679		isoorientina
6.564	BV	1	32.73198	0.00000	297.39540		vitexina
6.829	VB	1	223.29266	0.00000	295.37176		rutina
7.089	BB	1	43.27822	0.00000	291.12869		isovitexina
7.647	BB	1	44.89077	0.00000	302.19461		iperoside
15.290	BB	1	20.97645	0.00000	303.48503		quercetina
18.785		1	-	-	-		kamferolo

Totals without ISTD(s) : 1789.00227

2 Warnings or Errors :

Warning : Calibration warnings (see calibration table listing)
Warning : ISTD compound(s) not found

==
*** End of Report ***
