# Supplementary figures and images for: Buckwheat Hull Extracts Inhibit Aspergillus flavus Growth and AFB1 Biosynthesis
Source: Front Microbiol. 2019 Aug 29;10:1997. doi: 10.3389/fmicb.2019.01997 (PMC6727613; doi:10.3389/fmicb.2019.01997)

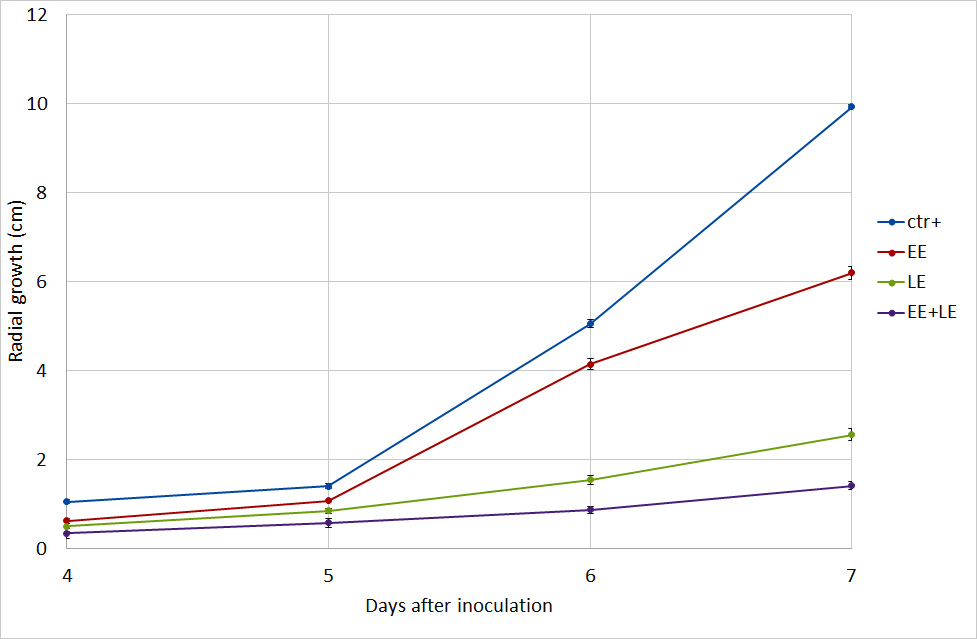

Supplement: FIGURE S2 — Fungal growth (cm) in cultural medium amended with phenolic extract (PE) and lipophilic extract (LE), monitored at 4, 5, 6, and 7 days after inoculation (DAI). Positive control (ctr+) corresponded, respectively, to non-inoculated growth medium amended with extracts, and infected growth medium without extracts. Values represented the mean of n = 5 determinations ± SE. [file Image_2.jpeg]

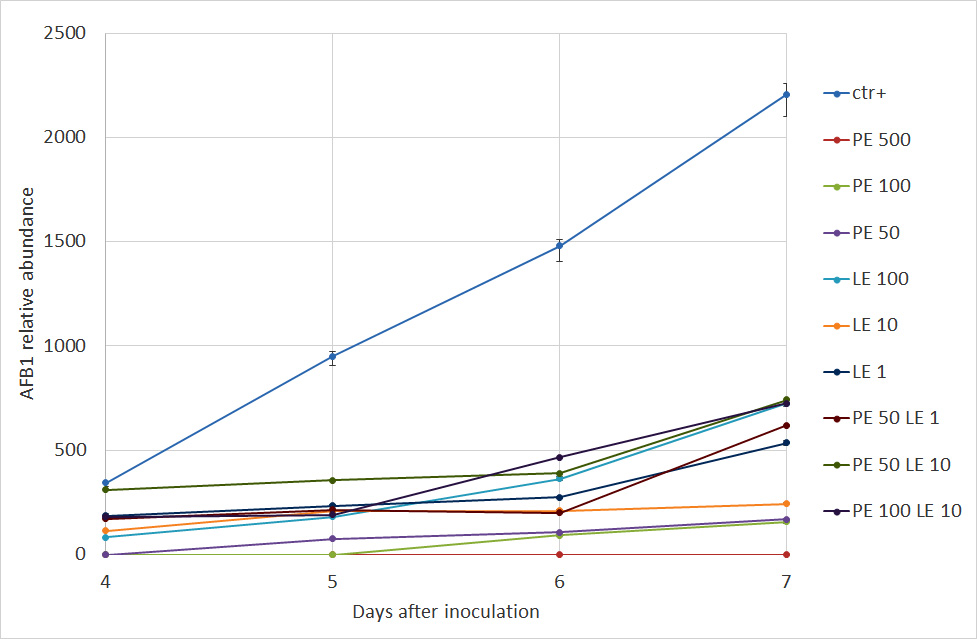

Supplement: FIGURE S3 — AFB1 biosynthesis in cultural medium amended with phenolic extract (PE) and lipophilic extract (LE), alone or in combinations of a range of concentrations (indications provided in the graph legend) monitored at 4, 5, 6, and 7 days after inoculation (DAI). Positive control (ctr+) corresponded, respectively, to non-inoculated growth medium amended with extracts, and infected growth medium without extracts. Values represented the mean of n = 5 determinations ± SE. [file Image_3.jpeg]
